# From Radioactive Effluent to Drinking Water: Efficient Removal of Trace ^99^TcO_4_^−^/ReO_4_^−^ by Cationic Porous Aromatic Framework

**DOI:** 10.1002/advs.202414604

**Published:** 2025-01-14

**Authors:** Long‐Sheng Pang, Xiangjun Liao, Chao‐Yue Zhao, Cheng‐Peng Li, Zhong Liu, Shengqian Ma

**Affiliations:** ^1^ College of Chemistry Tianjin Key Laboratory of Structure and Performance for Functional Molecules Tianjin Normal University Tianjin 300387 China; ^2^ Ningbo Key Laboratory of Agricultural Germplasm Resources Mining and Environmental Regulation College of Science and Technology Ningbo University Ningbo Zhejiang 315300 China; ^3^ Key Laboratory of Comprehensive and Highly Efficient Utilization of Salt Lake Resources Qinghai Provincial Key Laboratory of Resources and Chemistry of Salt Lakes Qinghai Institute of Salt Lakes Chinese Academy of Sciences Xining Qinghai 810008 China; ^4^ Department of Chemistry University of North Texas1508 W Mulberry St Denton TX 76201 USA

**Keywords:** deep purification, ionic liquids, perrhenate, pertechnetate, porous aromatic framework

## Abstract

Efficient removal of ^99^TcO_4_^−^ from radioactive effluents while recovering drinking water remains a challenge. Herein, an excellent ReO_4_^−^ (a nonradioactive surrogate of ^99^TcO_4_^−^) scavenger is presented through covalently bonding imidazolium poly(ionic liquids) polymers with an ionic porous aromatic framework (iPAF), namely iPAF‐P67, following an adsorption‐site density‐addition strategy. It shows rapid sorption kinetics, high uptake capacity, and exceptional selectivity toward ReO_4_^−^. Notably, the residual concentration of TcO_4_^−^/ReO_4_^−^ in the radioactive wastewater after iPAF‐P67 treatment is as low as 0.046 ppb, fully meeting the drinking water standards of World Health Organization (WHO, 0.159 ppb) and United States Environmental Protection Agency (U.S. EPA, 0.053 ppb). Density functional theory (DFT) calculations show that the imidazolium groups in iPAF‐P67 provide stronger electrostatic interactions and higher binding energies between iPAF‐P67 and TcO_4_^−^ anions, leading to its superior adsorption performance. Furthermore, the scale‐up synthesized iPAF‐P67 materials are shaped with polyethersulfone (PES) to fabricate PAF‐P67/PES beads and nanofibers via phase inversion method and electrospinning technique, respectively. Both composites demonstrate outstanding ultra‐purification abilities toward ReO_4_^−^ to meet the WHO criteria even after multiple dynamic adsorption/desorption cycles. This work develops a design strategy for adsorbents applicable in the sequestration of low‐concentration radioactive pollutants.

## Graphical abstract

Based on the adsorption‐site density‐addition strategy, an imidazolium‐enriched material (iPAF‐P67) is obtained from covalently bonding imidazolium ionic liquids to a ionic porous aromatic framework (iPAF‐67). Upon shaping, its beads and nanofibers demonstrate outstanding ultra‐purification abilities toward ReO_4_^−^/TcO_4_^−^ from nuclear wastewater, to meet the WHO and US EPA drinking water requirements.

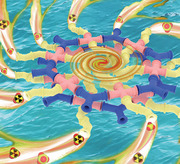

## 1 Introduction

Access to safe and affordable drinking water is recognized as a fundamental human right.^[^ ^1^ ^]^ The Sustainable Development Goal 6 (SDG 6) on water and sanitation, integral to the 2030 Agenda for Sustainable Development, explicitly stipulates universal access to this essential service by 2030.^[^ ^2^ ^]^ However, in recent decades, global concerns regarding radioactive contamination and the safety of drinking water have intensified due to nuclear accidents and the discharge of nuclear‐contaminated water.^[^ ^3^ ^]^ Notably, technetium‐99 (^99^Tc), originating from uranium fission reactors, poses significant radiation risks and environmental hazards due to its long half‐life (2.13 × 10^5^ years) and high fission yield (≈6%).^[^ ^4^ ^]^ During spent fuel reprocessing and waste disposal, the intricate chemical behavior of ^99^Tc complicates valence state control and the selective separation of uranium, neptunium, and plutonium. Additionally, the high volatility of ^99^Tc species (Tc_2_O_7_) limits effective sequestration through high‐temperature vitrification.^[^ ^5^ ^]^ More seriously, the high water solubility (existed in the form of ^99^TcO_4_^−^) and the nearly non‐complexing nature of ^99^Tc allow it to easily enter the ecosystem through multiple pathways, jeopardizing human health even at trace concentrations.^[^ ^6^ ^]^ Therefore, the U.S. EPA has established a maximum contaminant level (MCL) of 4 millirems per year for beta particle and photon radioactivity of man‐made radionuclides (including ^99^Tc) in drinking water.^[^ ^7^ ^]^ However, excessive ionic competition makes it difficult to achieve high removal efficiencies at extremely low radionuclide concentrations, ultimately ensuring safe drinking water.

To address this challenging issue, solid sorbents are considered promising materials to sequester TcO_4_^−^ due to their simplicity of preparation, controllable cost, excellent performance, and point‐of‐use applications.^[^ ^8^ ^]^ However, most commercialized polymeric anion‐exchange resins (e.g., Purolite‐A‐530E, SuperLig‐639, IRA‐401) exhibit slow anion exchange kinetics and poor radiation resistance.^[^ ^9^ ^]^ Despite the high selectivity and stability demonstrated by cationic polymer networks (CPNs) toward TcO_4_^−^, their applicability is constrained by the preparation conditions and product yields.^[^ ^10^ ^]^ For instance, the synthesis of cationic covalent organic frameworks (COFs) is challenging, and some exhibit inadequate alkali durability;^[^ ^11^ ^]^ only a few of cationic metal–organic frameworks (MOFs) are able to withstand the rigors of harsh working conditions due to the poor hydrolytic stability.^[^ ^12^ ^]^ Our group has enhanced the adsorption performance and stability of sorbents in harsh environments by post‐polymerizing ionic liquids (ILs) on MOFs and COFs.^[^ ^13^ ^]^ However, limited by the density of specific trapping sites on the adsorbent, this approach is still insufficient to achieve efficient capture of trace TcO_4_^−^ from ultra‐low concentrations in radioactive wastewater while realizing the recovery of drinking water. To this end, several key features should be considered as for the sorbent material: 1) possessing a stable rigid porous framework structure;^[^ ^14^ ^]^ 2) incorporating specific capture moieties for TcO_4_^−^; and 3) maximizing the density of moieties on the framework material.

**Scheme 1:**
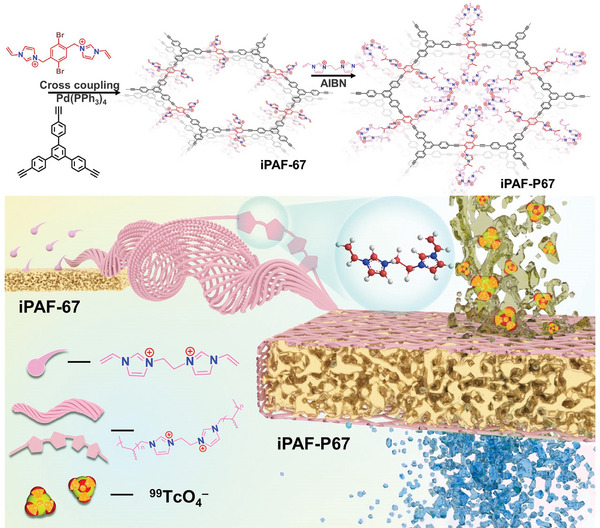
Schematic view of iPAF‐67 fabrication via the adsorption‐site density‐addition strategy.

Porous aromatic frameworks (PAFs) represent an advanced class of porous organic polymer materials synthesized via carbon–carbon coupling reactions between various rigid aromatic building units.^[^ ^15^ ^]^ The rigid porous framework endows PAFs with features such as large surface area and high porosity.^[^ ^16^ ^]^ Additionally, the specific functionality of PAFs can be modulated by selecting different building units or designing coupling reactions to form connecting struts. The stable carbon–carbon covalent bonds ensure the applicability of PAFs in harsh chemical environments (acidity, alkalinity, etc.).^[^ ^17^ ^]^ Notably, ionic PAFs (iPAFs) can bind to anions through electrostatic interactions and van der Waals forces. Although these characteristics make iPAFs the preferred adsorbents for capturing TcO_4_^−^, they are still insufficient for selective removal of ultra‐low concentrations of TcO_4_^−^ especially in the presence of multiple competing ions. To enhance the binding affinity between the adsorbent and TcO_4_^−^, it is crucial to explicitly incorporate functional groups that can selectively capture TcO_4_^−^. Our previous studies have shown that imidazolium plays a critical role in achieving highly selective removal of TcO_4_^−^.^[^ ^13^ ^]^ Furthermore, imidazolium usually exists in ILs, which can be impregnated into the pores of porous materials via in situ polymerization. Accordingly, we creatively propose an adsorption‐site density‐addition strategy via a two‐step fabrication process: 1) constructing functionalized iPAFs via cross‐coupling reactions with imidazolium‐based building units, and 2) increasing the density of imidazolium groups by further coating imidazolium‐based ILs onto the functionalized iPAF (**Scheme** **1**). Overall, we are committed to achieving the efficient removal of TcO_4_^−^ from low‐concentration radioactive wastewater and simultaneously producing drinking‐grade purified water by synthesizing adsorbents with rigid porous frameworks and ultra‐high imidazolium densities.

Hence, in this work, a poly(ionic liquid)‐modified iPAF (polyILs@iPAF, denoted as iPAF‐P67) based on an adsorption‐site density‐addition strategy was synthesized (Scheme 1). A vinylimidazolium‐functionalized precursor was employed to form iPAF‐67, which was further polymerized with vinylimidazolium ILs to yield iPAF‐P67 composite. Consequently, iPAF‐P67 possesses an exceptionally high density of imidazolium and exhibits superior removal efficiency for ultra‐low concentrations of ReO_4_^−^ (a nonradioactive surrogate of TcO_4_^−^)^[^ ^18^ ^]^ in nuclear wastewater. Notably, the residual TcO_4_^−^/ReO_4_^−^ concentration in water is as low as 0.046 ppb after iPAF‐P67 treatment, fully meeting the drinking water standards issued by the WHO (0.159 ppb) and U.S. EPA (0.053 ppb). To verify its practical industrial application, iPAF‐P67 powders were shaped with polyethersulfone (PES) into polymer beads and nanofibers through granulation and electrospinning methods, respectively. As a result, both iPAF‐P67@PES beads and nanofibers not only retained the excellent removal performance of the parent iPAF‐67 adsorbents but also exhibited remarkable durability and recyclability.

## 2 Results and Discussion

### 2.1 Synthesis and Structural Analysis of iPAF‐P67

As shown in Scheme 1, iPAF‐P67 with an extremely high density of imidazolium was prepared via a two‐step reaction. First, iPAF‐67 was synthesized via the Sonogashira‐Hagihara cross‐coupling reaction between the cationic unit 1‐({2,5‐dibromo‐4‐[(3‐vinylimidazol‐1‐ium‐1‐yl)methyl]phenylmethyl)‐3‐vinylimidazol‐1‐ium dibromide (DBVIB) and the triangular linker 1,3,5‐tris (4‐ethynylphenyl)benzene (TEPB). The resultant iPAF‐67 possessed a cationic porous framework and bilaterally pendent vinyl groups suitable for post‐polymerization. Subsequently, 3,3′‐divinyl‐1,1′‐(1,2‐ethanediyl)‐diimidazolium dibromide (*bis*‐C_2_), which contains two vinylimidazolium groups, was introduced to co‐polymerize with the pre‐synthesized iPAF‐67, resulting in iPAF‐P67. This two‐step reaction following the adsorption‐site density‐addition strategy successfully increased the amount of imidazolium groups within iPAF‐P67.

**Figure 1:**
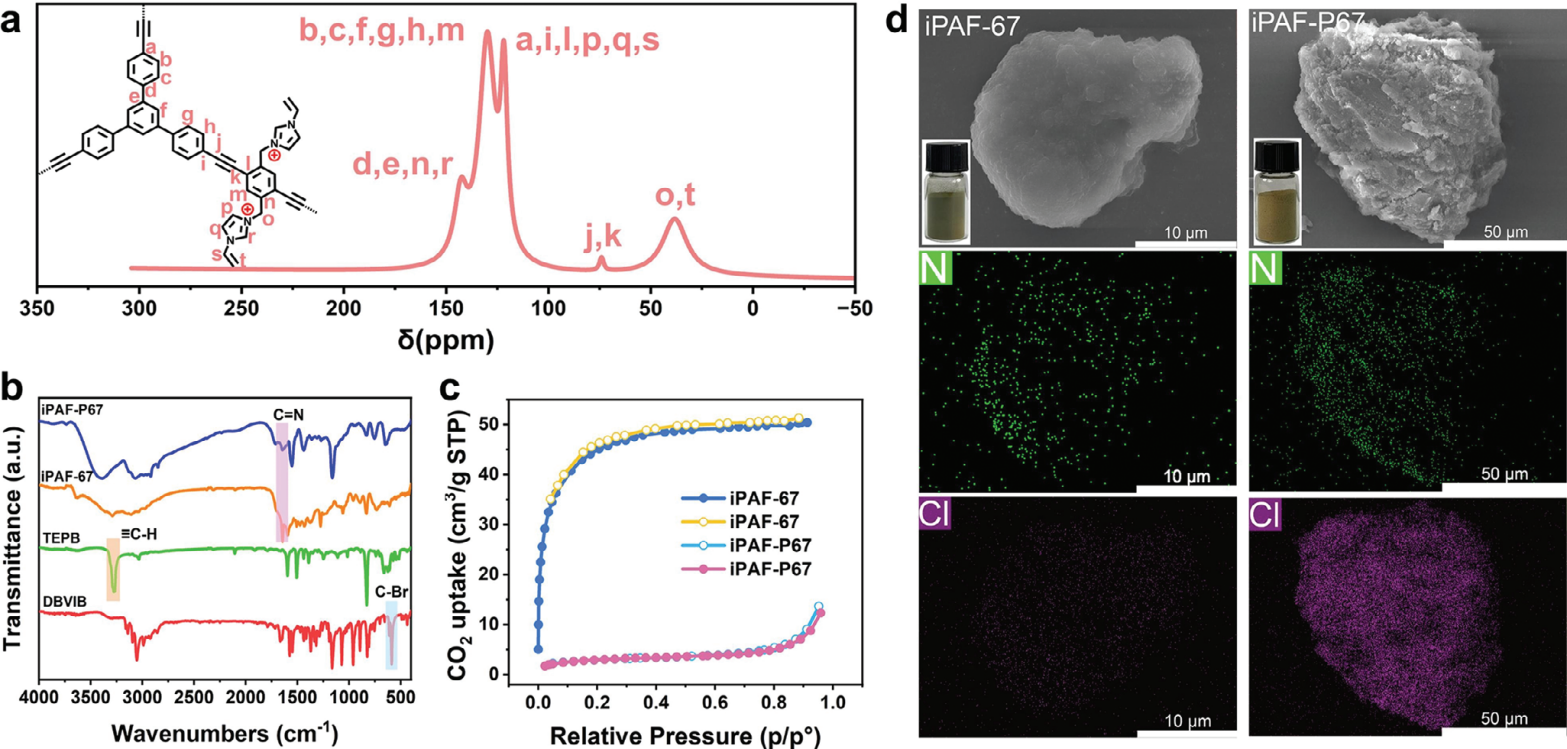
Syntheses and characterization of iPAF‐67 and iPAF‐P67. a) Solid‐state ^13^C NMR spectra of iPAF‐67. b) FTIR spectra of iPAF‐67, iPAF‐P67, TEPB, and DBVIB. c) CO_2_ adsorption–desorption isotherms for iPAF‐67 and iPAF‐P67. d) SEM micrographs and EDS mapping images of iPAF‐67 and iPAF‐P67 (bottom inset: gram‐level powder photographs).

The evolution of chemical moieties throughout the synthesis of iPAF‐P67 was comprehensively investigated. As depicted in **Figure** **1**a, cross‐polarization and magic angle spinning ^13^C solid‐state nuclear magnetic resonance (^13^C NMR) spectra elucidated the occurrence of a cross‐coupling reaction between DBVIB and TEPB to yield iPAF‐67. The characteristic peaks at 121.7 ppm and 142.5 ppm can be ascribed to the carbon atoms from the imidazolium rings.^[^ ^19^ ^]^ Fourier transform infrared spectroscopy (FTIR) analyses revealed the disappearance of the characteristic peaks of ≡C─H of TEPB at 3270 cm^─1^and C─Br of DBVIB within 500–600 cm^−1^ in the spectrum of iPAF‐67. Meanwhile, the C═N peaks (1630 and 1570 cm^−1^) of imidazolium appeared in the spectrum of iPAF‐67 and iPAF‐P67 (Figure 1b), demonstrating the presence of imidazolium groups therein.

The pore characteristics of the adsorbent material were determined by the CO_2_ adsorption–desorption isotherm at 198K (Figures 1c; Figure S1, Supporting Information). The Brunauer–Emmett–Teller (BET) surface areas of iPAF‐67 and iPAF‐P67 were analyzed to be 173.9 and 10.2 m^2^ g^−1^, respectively. This significant decrease in porosity can be attributed to the additional *bis*‐C_2_ polymers adhering to the framework of iPAF‐P67. To further verify the effectiveness of the adsorption‐site density‐addition strategy, scanning electron microscopy (SEM) and energy‐dispersive X‐ray spectroscopy (EDS) analyses were conducted on iPAF‐67 and iPAF‐P67. The iPAF‐67 particles, ≈20 µm in size, have a smooth surface, which expands to ≈100 µm for iPAF‐P67 particles with a rough surface (Figures 1d; Figure S2, Supporting Information). These iPAF‐67 and iPAF‐P67 powders can be facilely synthesized at the gram level (≈4 g per reaction) in the lab (Figure 1d). EDS mapping showed significantly higher concentrations of Cl and N elements in iPAF‐P67 compared to iPAF‐67, clearly demonstrating that the density of imidazolium groups in iPAF‐P67 is substantially higher than in iPAF‐67. Correspondingly, EDS elemental analyses revealed the concentrations of imidazolium groups are 1.54 and 4.57 mmol g^−1^ for iPAF‐67 and iPAF‐P67, respectively (Figure S3, Supporting Information). Moreover, ion chromatography (IC) data indicated that the halogen content in iPAF‐P67 was 17 times higher than that in iPAF‐67 (Figure S4 and Table S1, Supporting Information). These results imply that the imidazolium moiety density in iPAF‐P67 significantly increases after co‐polymerization, which is expected to improve the adsorption capacity and expedite the exchange rate toward TcO_4_^−^/ReO_4_^−^. Additionally, due to the impregnation of abundant imidazolium groups, the water contact angle of the solid decreased from 98° for iPAF‐67 to 49° for iPAF‐P67 (Figure S5, Supporting Information). This transition from hydrophobicity to hydrophilicity is anticipated to promote effective contact and rapid adsorption of the adsorbent with radionuclides in aqueous environments.^[^ ^20^ ^]^

### 2.2 ReO_4_^−^ Adsorption Performance of iPAF‐P67

**Figure 2:**
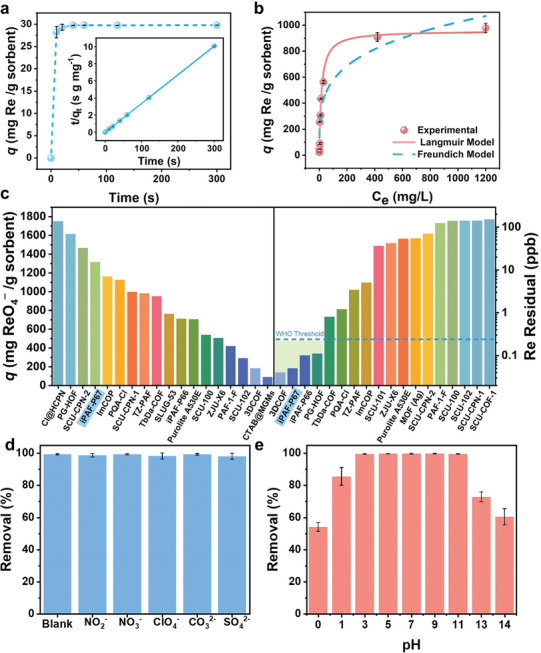
Adsorption performances of iPAF‐P67. a) Adsorption kinetics of iPAF‐P67 toward ReO_4_^−^ and its pseudo‐second‐order linear fitting plots (Inset). b) Adsorption isotherm of iPAF‐P67 for ReO_4_^−^ uptake. c) Comparison of the maximum ReO_4_^−^ adsorption capacity and minimum ReO_4_^−^ residue between IPAF‐P67 and reported adsorbents. d) Removal efficiency of ReO_4_^−^ by iPAF‐P67 in the presence of competitive anions. (e) Effect of pH on the ReO_4_^−^ sorption of iPAF‐67.

The adsorption performances of iPAF‐P67 toward TcO_4_^−^ were investigated by adsorption kinetics and adsorption thermodynamics experiments. Specifically, the adsorption kinetics of iPAF‐P67 was observed by introducing 5 mg of iPAF‐P67 powder into 10 mL of aqueous solution containing 25 ppm ReO_4_^−^. As depicted in **Figure** **2**a, iPAF‐P67 demonstrated remarkable adsorption kinetics, achieving equilibrium (*q*_e_ = 29.81 mg g^−1^) and removing 99.35% of ReO_4_^−^ from the solution within 1 min. The ReO_4_^−^ adsorption kinetic data were highly consistent with a pseudo‐second‐order kinetic model (correlation coefficient *R* ^2^ > 0.9999), and the rate constant *K*_2_ reached 10.13 g mg^−1^ min^−1^ (Table S2, Supporting Information), which is much higher than that of commercial anion‐exchange resins and comparable porous organic framework materials.^[^ ^21^ ^]^ Furthermore, the distribution coefficient (*K*_d_) was employed to quantitatively evaluate the affinity of the adsorbents for radionuclides. The *K*_d_ value of iPAF‐P67 (1.1 × 10^6^ mL g^−1^) exceeds the exceptional assessment criterion (1.0 × 10^5^ mL g^−1^) by an order of magnitude, indicating its ultra‐high affinity for TcO_4_^−^/ReO_4_^−^. Subsequently, the adsorption isotherm of iPAF‐P67 was examined by exposure to ReO_4_^−^ aqueous solutions at concentrations ranging from 50 ppm to 3000 ppm to gain further insights into the adsorption properties of the synthesized materials. The maximum adsorption capacity from the Langmuir model for iPAF‐P67 is 1317 mg g^−1^ (Figure 2b; Table S3, Supporting Information), surpassing that of commercial resins such as Purolite A532E (446 mg g^−1^)^[^ ^22^ ^]^ and Purolite A530E (706 mg g^−1^),^[^ ^23^ ^]^ as well as some outstanding porous adsorbents such as TZ‐PAF (982 mg g^−1^)^[^ ^24^ ^]^ and SCU‐CPN‐1 (997 mg g^−1^).^[^ ^6^ ^]^ This value is only lower than that of SCU‐CPN‐2 (1476 mg g^−1^),^[^ ^25^ ^]^ PG‐HOF‐1 (1616 mg g^−1^),^[^ ^26^ ^]^ and Cl@HCPN (1752 mg g^−1^).^[^ ^27^ ^]^ Meanwhile, the adsorption capacity of iPAF‐P67 is significantly enhanced compared to iPAF‐67 (478 mg g^−1^), due to the introduction of *bis*‐C_2_ conferring more functional groups (Figure S6 and Table S3, Supporting Information).

The efficient adsorption of target nuclides from low‐concentration radioactive solutions is both challenging and pressing for various application scenarios. For instance, the inadvertent release of even trace ^99^Tc, known for its high mobility in water, can cause enduring ramifications on the ambient ecosystem. According to publicly available data from Tokyo Electric Power Company, the activity levels of ^99^Tc (72 Bq L^−1^) in wastewater storage tanks exceed normal screening thresholds, despite rigorous maintenance at attenuated concentrations. Therefore, our endeavor aimed to deploy the developed iPAF‐P67 for the sequestration of TcO_4_^−^/ReO_4_^−^ at ultra‐low concentrations. Utilizing ReO_4_^−^‐infused solutions at 25 ppb, the removal percentage of ReO_4_^−^ by iPAF‐P67 was above 99.8%. Consequently, the residual concentrations in the test solutions were reduced to a mere 0.046 ppb (Figure 2c), aligning with the stipulated drinking water quality benchmarks outlined by the WHO (<0.159 ppb) and surpassing even the stringent standards promulgated by the U.S. EPA (< 0.053 ppb).^[^ ^7^ ^]^ Notably, while a few adsorbents have demonstrated significant advantages in uptake capacities of TcO_4_^−^/ReO_4_^−^ (> 1 g·g^−1^) (Figure 2c),^[^ ^24^, ^25^, ^26^, ^27^, ^28^ ^]^ their utility in nuclear wastewater treatment is limited because none of them can reduce the radionuclide residual below drinking water thresholds. In contrast, iPAF‐P67 demonstrates both a high uptake capacity (1317 mg g^−1^), fast kinetics (< 1 min), and extremely low residual emission (0.046 ppb), an attribute hitherto unattained by existing materials.

Given the presence of excessive competing anions in the radioactive waste stream, the preferential selectivity of the adsorbent material for the TcO_4_^−^/ReO_4_^−^ needs to be verified.^[^ ^29^ ^]^ As shown in Figure 2d, iPAF‐P67 demonstrates a remarkable capability to trap ≈100% of ReO_4_^−^ in the presence of competing anions such as NO_2_^−^, NO_3_^−^, ClO_4_^−^, CO_3_^2−^, SO_4_^2−^. Additionally, we explored the feasibility of iPAF‐P67 adsorption for two prototypical radioactive solutions encountered in real‐world scenarios (Tables S4 and Figure S5, Supporting Information). The results indicated that iPAF‐P67 achieved a ReO_4_^−^ removal percentage of 89.44% in Hanford low activity waste (LAW) and 60.74% in simulated legacy nuclear wastes in Savannah River Site (SRS), surpassing most reported PAF adsorbents.^[^ ^21^, ^24^ ^]^ The superior selectivity of iPAF‐P67 for TcO_4_^−^/ReO_4_^−^ can be attributed to the strong affinity between TcO_4_^−^/ReO_4_^−^ and imidazolium moieties.^[^ ^30^ ^]^ The positively charged imidazolium group engenders electrostatic and additional interactions (*p*–*p* and *p*−*π*) with TcO_4_^−^/ReO_4_^−^, thereby facilitating the selective sequestration of these species.^[^ ^31^ ^]^

Moreover, the stability of the adsorbent material under harsh acidic and alkaline conditions, alongside its recyclability, is paramount to meet the requirements of practical applications. To evaluate its acid and alkali resistance, iPAF‐P67 was immersed in ReO_4_^−^ solutions of pH 0–14 (Figure 2e). In the range of pH 1–13, iPAF‐P67 maintained more than 90% ReO_4_^−^ removal. However, in 1 M HNO_3_ or 1 M NaOH (NO_3_^−^/OH^−^:ReO_4_^−^ = 10 000:1), the removal efficiency decreased to 54% or 65%, respectively. Regarding recyclability, ReO_4_^−^‐loaded iPAF‐P67 was desorbed by immersion in the saturated NaCl solution, followed by the repetition of the adsorption–desorption process. Remarkably, even after undergoing five consecutive cycles, iPAF‐P67 still demonstrated a removal percentage of more than 99% for ReO_4_^−^ (Figure S7, Supporting Information). Evidently, the advantages of iPAF‐P67 hold profound significance in mitigating emergency accidents and remediating irreversible ecological crisis.

### 2.3 Adsorption Mechanism of ReO_4_^−^

**Figure 3:**
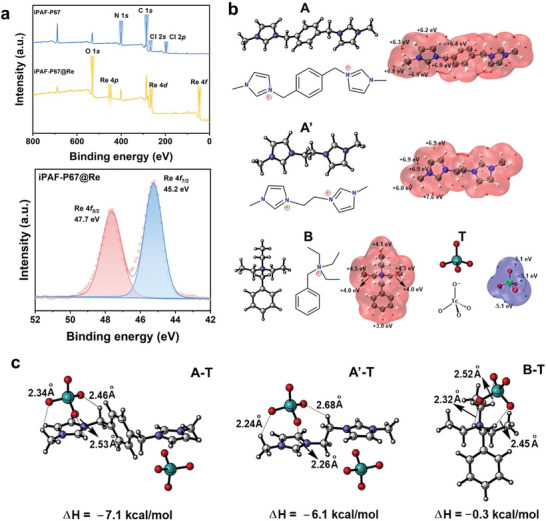
Mechanisms of ReO_4_^−^/TcO_4_^−^ sorption by iPAF‐P67. a) XPS survey spectra of iPAF‐P67 and iPAF‐P67@Re (upper), and Re 4*f* survey of iPAF‐P67@Re (bottom). b) ESP distributions on the van der Waals surfaces of A and A’ (representative fragments of iPAF‐P67), B (representative fragments of commercial resins, e.g. Purolite A530E), and T (TcO_4_^−^ /ReO_4_^−^). (c) Optimized geometries of A‐T, A′‐T, and B‐T. The hydrogen bonding distance between atoms (Å) and the adsorption enthalpy (∆*H*) between the target element and the adsorption material are labeled.

To elucidate the sorption mechanism, X‐ray photoelectron spectroscopy (XPS) and density functional theory (DFT) calculations were combined to examine the interactions between iPAF‐P67 and ReO_4_^−^/TcO_4_^−^. Compared to iPAF‐P67, the spectrum of iPAF‐P67@Re exhibits Re 4*p*, Re 4*d*, and Re 4*f* peaks, while the Cl 2*p* peak nearly disappears due to ion exchange (**Figure** **3**a). Analysis of the Re 4*f* core‐level spectrum showed a red shift in the binding energy of Re 4*f*_7/2_ from 48.1 eV in KReO_4_ to 45.2 eV in iPAF‐P67@Re, suggesting an increase in electron density due to the interaction between the N^+^ donor of imidazolium groups in iPAF‐P67 and ReO_4_^−^/TcO_4_^−^.^[^ ^19^, ^32^ ^]^

Additionally, DFT results provide deeper insights into the superior adsorption capabilities of iPAF‐P67 for TcO_4_^−^ compared to commercial resins, Purolite A530E. The optimized structures and electrostatic potential distributions of the iPAF‐P67 fragments (A and Aʹ), and Purolite A530E fragment (B) are shown in Figure 3b. Notably, the maximum positive potential in fragments A and Aʹ is located near the imidazole ring, ≈+6.9 eV, whereas in fragment B, the maximum potential near the ethyl group is around + 4.5 eV. This indicates that the imidazolium groups in A and A' of iPAF‐P67 are more predisposed to capture negatively charged TcO_4_^−^ (−5.1 eV) via electrostatic attraction. Furthermore, the adsorption enthalpy (Δ*H*), which reflects the affinity between the anion and the adsorbent, was calculated. Upon capturing TcO_4_^−^, the optimized segments of iPAF‐P67 and Purolite A530E show that TcO_4_^−^ anion is located above the positive sites of imidazolium and quaternary ammonium, respectively, and C─H─O hydrogen bonds form (Figure 3c). The Δ*H* values for A‐T (−7.1 kcal mol^−1^) and A'‐T (−6.1 kcal mol^−1^) are significantly higher than for B‐T (−0.3 kcal mol^−1^). These results clearly demonstrate that iPAF‐P67 is more favorable to sequester TcO_4_^−^ than quaternary ammonium‐type commercial resins (e.g., Purolite A530E).

### 2.4 Adsorption Applications of iPAF‐P67/PES Beads and Nanofibers

**Figure 4:**
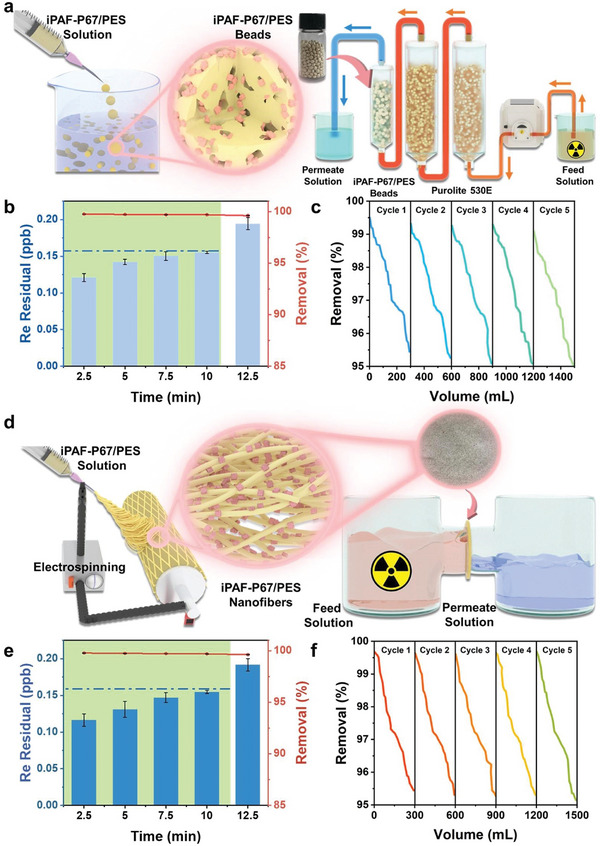
Preparation and performance of iPAF‐P67/PES beads and nanofibers. a) Schematic illustration of iPAF‐P67/PES beads fabrication and laboratory‐built continuous flow system. b) Dynamic adsorption of ReO_4_^−^ by IPAF‐P67/PES beads. The blue dashed line indicates the threshold concentration (0.159 ppb) of the WHO drinking water guideline. c) ReO_4_^−^ removal efficiency of iPAF‐P67/PES beads in five consecutive adsorption–desorption cycles. d) Schematic illustration of iPAF‐P67/PES nanofibers fabrication and gravity‐driven H‐type membrane cell. (e) Dynamic adsorption of ReO_4_^−^ by IPAF‐P67/PES nanofibers. The blue dashed line indicates the threshold concentration (0.159 ppb) of the WHO drinking water guideline. (f) ReO_4_^−^ removal efficiency of iPAF‐P67/PES nanofibers in five consecutive adsorption–desorption cycles.

Encouraged by the results above, we then were motivated to shape iPAF‐67 into resins and membranes, which are considered effective methods to overcome the inconvenience of adsorbent powder in large industrial reactors from a processing perspective. Therefore, iPAF‐P67 powders were shaped with hydrophobic PES as the substrate into polymer beads and nanofibers through granulation and electrospinning methods, respectively. The iPAF‐P67/PES beads were prepared by employing the immersion precipitation technique. As shown in **Figure** **4**a, the process involved the sequential extrusion of a well‐mixed iPAF‐P67/PES solution through a syringe, followed by immersion in water to induce solvent‐nonsolvent exchange, thereby facilitating phase inversion. Consequently, iPAF‐P67 was uniformly dispersed within a multilayer structure of iPAF‐P67/PES beads, each with an approximate diameter of ≈3.30 mm (Figures S8 and S9, Supporting Information). To explore the dynamic adsorption performance of iPAF‐P67/PES beads targeting ReO_4_^−^ in a low‐concentration radioactive stream, a laboratory‐built continuous flow system was established (Figure 4a). Specifically, a column containing 1.5 g of iPAF‐P67/PES resin beads was connected behind two columns containing 200 g of Purolite A530E each. The concentration of the initial feed solution was 22.5 ppm ReO_4_^−^, and it remained above 50 ppb after passing through the two Purolite A530E columns, due to the limited in‐depth purification abilities of commercial resins. Subsequently, the effluent containing 50 ppb ReO_4_^−^ was pumped into the column filling with 200 g of Purolite A530E or 1.5 g of iPAF‐P67/PES bead, respectively. Even at such a low feed concentration, the iPAF‐P67/PES beads column demonstrated remarkable efficiency in ReO_4_^−^ removal (> 99%). The ReO_4_^−^ concentration maintained in the range of 0.127–0.155 ppb within a 10 min period (Figure 4b), being lower than the threshold concentration of WHO drinking water guideline (0.159 ppb). In comparison, the removal percentage of ReO_4_^−^ treated by Purolite A530E was only ca. 36% and the residual ReO_4_^−^ concentration was ca. 32–36 ppb within a 10 min period. This result demonstrated the deep purification capacity of iPAF‐P67/PES was significantly stronger than that of Purolite A530E. Moreover, the reusability of iPAF‐P67/PES beads was validated. The iPAF‐P67/PES reins column maintained more than 95.05% ReO_4_^−^ removal after five adsorption–desorption cycles (1500 mL effluent) (Figure 4c). Notably, the morphologies of iPAF‐P67/PES beads remained unchanged during the recycling test (Figures S8 and S9, Supporting Information), underscoring their robust performance in industrial production.

In addition, we extended our investigation by fabricating iPAF‐P67/PES nanofibers via electrospinning, aiming to explore its universality across diverse carriers. As depicted in Figure 4d, the polymer solution within the syringe underwent ejection in the form of tiny jets propelled by electric field forces, subsequently undergoing continuous stacking and solidification on the receiving drum, ultimately yielding the iPAF‐P67/PES nanofibers. Subsequently, the as‐developed iPAF‐P67/PES nanofibers were sandwiched on an H‐shaped test cell, and the adsorption test process was run under gravity drive (Figure 4d). The results showed that the low concentration of ReO_4_^−^ solution (50 ppb) on the feed side was reduced to 0.123 ppb after permeation through the iPAF‐P67/PES nanofibers, meeting the requirement of WHO drinking water criteria (Figure 4e). Moreover, removal of ReO_4_^−^ by iPAF‐P67/PES nanofibers still exceeded 95.14% after five consecutive adsorption–desorption cycles (Figure 4f). This underscores the remarkable versatility of iPAF‐P67 with superior ReO_4_^−^ adsorption capabilities across various carriers, thus promising a potential application in diverse domains.

## 3 Conclusion

In conclusion, we propose an adsorption‐site density‐addition strategy for the development of an excellent ^99^TcO_4_^−^/ReO_4_^−^ a scavenger of iPAF‐P67 through covalently bonding imidazolium ILs polymers with iPAF‐67, yielding clean water that meets internationally recognized drinking water standards. The ultrahigh density of imidazolium‐specific moieties endowed iPAF‐P67 with excellent performances in treating Tc/Re‐containing stream, including simulated Hanford LAW and SRS high‐level nuclear waste, as supported by DFT calculations. Moreover, shaping as‐developed iPAF‐P67/PES into polymer beads and nanofibers evidently verified the tangible prospects of such a design strategy for practical implementation, including but not limited to emergency disposal of radioactive waste leakage. Our endeavors not only culminate in the creation of an adsorbent matrix for the capture of low‐concentration radioactive contaminants but also innovatively propose a design paradigm for adsorbents with broader applicability across water treatment domains.

## Conflict of Interest

The authors declare no conflict of interest.

## Supporting information



Supporting Information

## Data Availability

The data that support the findings of this study are available from the corresponding author upon reasonable request.
