# Supplementary material for: From Radioactive Effluent to Drinking Water: Efficient Removal of Trace 99TcO4 −/ReO4 − by Cationic Porous Aromatic Framework
Source: Adv Sci (Weinh). 2025 Jan 14;12(9):2414604. doi: 10.1002/advs.202414604 (PMC11884533; doi:10.1002/advs.202414604)
Supplement: Supplementary file 1 — Supporting Information [file ADVS-12-2414604-s001.pdf]

ADVANCED SCIENCE
Open Access

Supporting Information

for *Adv. Sci.*, DOI 10.1002/advs.202414604

From Radioactive Effluent to Drinking Water: Efficient Removal of Trace $^{99}TcO_4^-/ReO_4^-$ by Cationic Porous Aromatic Framework

Long-Sheng Pang, Xiangjun Liao, Chao-Yue Zhao, Cheng-Peng Li, Zhong Liu* and Shengqian Ma**

Supporting Information

From Radioactive Effluent to Drinking Water: Efficient Removal of Trace $^{99}TcO_4^-$ /ReO_4^- by Cationic Porous Aromatic Framework

Long-Sheng Pang, Xiangjun Liao, Chao-Yue Zhao, Cheng-Peng Li, Zhong Liu,* and Shengqian Ma**

Section S1. Experimental Section

1. Materials and chemicals

All materials and reagents were used directly unless otherwise stated. 1,4-dibromo-2,5-bis(bromomethyl)benzene and 1-vinylimidazole were purchased from Shanghai Bide Pharmatech Ltd. N, N-dimethylformamide (DMF) and triethylamine were provided by Chemart Ltd. 1,3,5-tris (4-ethylphenyl) benzene was purchased from Shanghai Bide Pharmatech Ltd. Tetrakis(triphenylphosphine)palladium was purchased from Beijing J&K Scientific Ltd. Cuprous iodide was supplied by Shanghai Macklin Biochemical Technology Co., Ltd. Azobis(diisobutyronitrile) (AIBN), methanol, polyethersulfone (PES) and polyethylene glycol (PEG) were provided by Sigma-Aldrich Ltd. $KReO_4$ was purchased from TCI (Shanghai) Development Co., Ltd.

2. Synthesis

Synthesis of 1-({2,5-dibromo-4-[(3-vinylimidazol-1-ium-1-yl) methyl] phenyl} methyl)-3-vinylimidazol-1-ium dibromide (DBVIB). 1,4-dibromo-2,5-bis(bromomethyl)benzene (2 mmol, 0.8435 g) and 1-vinylimidazole (6 mmol, 0.5646 g) were dissolved in acetonitrile (100 mL) and heated to 90°C under a N_2 atmosphere for 12 hours. After cooling the reaction mixture to room temperature, it was washed with acetonitrile. Finally, the product was filtered and vacuum-dried to obtain the solid DBVIB. Yield: 98%. ^{1}H NMR (600 MHz, d6-DMSO): δ (ppm) 9.58 (s, 1H), 8.29 (s, 1H), 7.91 (d, 3H), 7.34 (dd, 2H), 6.00 (dd, 2H), 5.56 (s, 3H), 5.46 (dd, 2H).

Synthesis of iPAF-67. Anhydrous N, N-dimethylformamide (15 mL) and triethylamine (15 mL) were added to a mixture containing DBVIB (1.5 mmol, 0.9500 g), 1,3,5-tris (4-ethylphenyl) benzene (1.5 mmol, 0.5678 g), tetrakis(triphenylphosphine)palladium (60 mg), and cuprous iodide (20 mg). The reaction mixture was heated to 80°C under a N_2 atmosphere for 72 hours. After cooling the reaction system to room temperature, the resulting solid was repeatedly washed with N, N-dimethylformamide, distilled water, and methanol. The solvents used are pre-filled with nitrogen. After centrifugation, the upper layer of clear liquid was removed to obtain a solid powder product. Finally, the product was subjected to Soxhlet extraction with tetrahydrofuran and acetone, followed by filtration and vacuum drying to obtain iPAF-67. Yield: 96%.

Synthesis of iPAF-P67. Firstly, iPAF-67 (250 mg) and bis-C_2 (1 g) were mixed with methanol (30 mL) at room temperature and stirred under N_2 atmosphere for 24 hours. Then, a methanol solution (2.5 mL) of azobis(diisobutyronitrile) (AIBN, 125 mg) was injected into the reaction system to initiate the polymerization by heating to 70 °C for 24 h under N_2 atmosphere. An

equal amount of AIBN was injected again and the reaction was continued at 70 °C for 24 hours. After cooling to room temperature, the solid product was washed with methanol, followed by filtration and vacuum drying to obtain iPAF-P67. Yield: 90%.

Synthesis of iPAF-P67 beads. The homogeneous polymer solution was prepared by dissolving iPAF-P67 (0.50 g), PES (1.20 g) and PEG (1.00 g) in DMF (5.00 g) by heating the mixture at 60°C. The iPAF-P67/PES beads were then obtained by dropping the polymer solution into a coagulation bath ($V_{water}/V_{ethanol}$ = 1:1) using a syringe.

Synthesis of iPAF-P67 nanofibers. Firstly, the electrospinning solution was prepared by dissolving iPAF-P67 (0.75 g), PES (1.75 g), and PEG (1.00 g) in DMF (7.50 g) with stirring and heating (60°C). The electrospinning process employed two 5 mL syringes, with the voltage set at 20-23 kV, a collection drum-to-needle distance of 15 cm, and a flow rate of 1.0 mL/h.

3. Characterizations

Solid-state ^{13}C cross-polarization magic angle spinning nuclear magnetic resonance (CP/MAS NMR) measurement was performed on a Bruker Avance Neo 400WB model 400 MHz NMR spectrometer. The ^{1}H NMR spectra were obtained using a Bruker AV 400 spectrometer, with chemical shifts reported in ppm relative to reference standards. FTIR spectra were recorded on a Bruker ALPHA FTIR spectrometer, employing KBr pellets within the range of 4000–400 cm^{-1}. Scanning transmission electron microscopy (STEM) and energy-dispersive X-ray spectroscopy (EDS) were conducted on a Thermo Fisher Scientific Talos F200X, operating at an electron beam energy of 200 keV. X-ray photoelectron spectroscopy (XPS) measurements were carried out on a Thermo Scientific ESCALAB 250Xi spectrometer. The Brunauer–Emmett–Teller (BET) surface areas of the adsorbent was determined by the CO_2 adsorption-desorption isotherm at 198K using Micromeritics ASAP 2020. Ion chromatography (ICS-1100) was utilized to measure halogen content in adsorbents. The in-air water contact angle (CA) was measured using an optical system (JY-82C) with a 5 μL droplet. The concentrations of ReO_4^- were determined using a Perkin-Elmer NexION 350D inductively coupled plasma mass spectrometry (ICP-MS).

4. Adsorption Tests

Batch experiments. In a typical batch experiment, the solid-to-liquid ratio was set at 2 $g \cdot L^{-1}$ by adding 10 mg of iPAF-67 or iPAF-P67 to 5 mL of aqueous solutions with varying concentrations of ReO_4^-. After stirring for the required time, the samples were separated using a 0.22 μm nylon membrane filter and diluted with 1% (w/w) HNO_3 to meet ICP-MS analysis requirements. A standard curve with a linear correlation coefficient greater than 0.999 was constructed using ReO_4^- solutions at concentrations of 0, 40, 50, 60, and 80 ppb. The removal

percentage of ReO_4^- (%), adsorption equilibrium capacity (q_e), and distribution coefficient (k_d) were calculated according to equations (1), (2), and (3)[1].

$$\text{Removal percentage (\%)} = \frac{c_0 - c_e}{c_0} \times 100\% \quad (1)$$

$$q_e = \frac{c_0 - c_e}{m} \times V \quad (2)$$

$$k_d = \frac{c_0 - c_e}{c_e} \times \frac{V}{m} \quad (3)$$

Where c_0 (mg·g^{-1}) and c_e (mg·g^{-1}) represent the initial and equilibrium concentrations of ReO_4^- in the sample solutions, respectively. V (mL) is the total volume of the solution, and m (g) is the mass of the adsorbent. In static adsorption tests, the simulated solution was filtered through a 0.22 μm aqueous filter membrane to remove solid impurities.

Sorption kinetics study. The ReO_4^- adsorption kinetics of iPAF-P67 was studied by ion-exchange experiments with different contact times. We added 10 mg of powder iPAF-P67 to 5 mL of solution containing 25 ppm ReO_4^-, and the mixture was magnetically stirred to achieve the desired contact time. The experimental dynamic data are analysed by using a pseudo-second-order dynamic model. The linearized form of the model is as follows[2]:

$$\frac{t}{q_t} = \frac{t}{q_e} + \frac{1}{k_2 q_e^2} \quad (4)$$

Where q_t (mg·g^{-1}) and q_e (mg·g^{-1}) represent the ReO_4^- adsorption capacities at time t and at equilibrium, respectively. k_2 (g·mg^{-1}·min^{-1}) is the pseudo-second-order rate constant.

Sorption isotherm investigations. The sorption isotherm experiments were conducted with the ReO_4^- concentrations ranging from 50 to 3000 ppm by adding iPAF-P67 (10 mg) to sample solution (5 mL) with specific concentrations. The Langmuir and Freundlich models were employed to elucidate the adsorption process. The Langmuir model assumes monolayer adsorption of ReO_4^- on a homogeneous surface, where the binding sites are equivalent with constant adsorption energies and no interaction between adsorbed ions. In contrast, the Freundlich model is based on a heterogeneous surface, where the isotherm assumes that the adsorbent surface sites have varying binding energies. The linear equations for the Langmuir and Freundlich isotherm models are expressed as follows[3]:

$$\frac{c_e}{q_e} = \frac{1}{q_m k_L} + \frac{c_e}{q_m} \quad (5)$$

$$\ln q_e = \ln k_F + \frac{1}{n} \ln c_e \quad (6)$$

where q_e (mg·g^{-1}) and c_e (mg·L^{-1}) represent the adsorption capacity and concentration at equilibrium, respectively. q_m (mg·g^{-1}) is the maximum adsorption capacity of the adsorbent. k_L is a constant indirectly related to the adsorption capacity and adsorption energy. k_F and n are the Freundlich constants related to adsorption capacity and adsorption intensity, respectively.

Anion-exchange competition study. To test the competitive effects of anions such as SO_4^{2-}, NO_2^-, NO_3^-, CO_3^{2-} and ClO_4^-, ion exchange experiments were conducted under specific solid-to-liquid ratio (1 g·L^{-1}), room temperature (~25 °C) and contact time (12 h). The sample solutions were prepared by adding 0.5 mM Na_2SO_4, $NaNO_2$, $NaNO_3$, Na_2CO_3, and $NaClO_4$ to a 0.5 mM ReO_4^- solution, respectively.

pH effect study. The pH of the sample solution was adjusted by adding dilute nitric acid or sodium hydroxide. 10 mg of iPAF-P67 was added to 5 mL of aqueous solution containing 25 ppm ReO_4^- with pH values ranging from 0 to 14. After stirring for 12 hours, the mixture was filtered using a 0.22 μm nylon membrane filter and analysed by ICP-MS.

Exchange experiments with simulated waste system. The simulated Hanford Low Activity Waste (LAW) and simulated high-level waste at Savannah River Site (SRS-HLW) streams were prepared according to reported protocols, respectively[4, 5]. For LAW, we added 10 mg of iPAF-P67 to 5 mL of simulated waste. For SRS HLW, adsorption tests were carried out at a solid-liquid ratio of 20 g·L^{-1}. The two sample solutions were stirred for 12 h, and the suspensions were separated by 0.22 μm nylon membrane filter for further determination by ICP-MS.

Reusability study. During a typical adsorption-desorption cycle, 10 mg of adsorbent material was immersed in 5 mL of ReO_4^- aqueous solution (25 ppm, pH = 7) stirred or run dynamically in a continuous test setup. Afterwards, the saturated adsorbent was placed in the saturated NaCl desorption solution and stirred for 12 h to promote ion exchange. The refresh adsorbent was again placed in ReO_4^- containing aqueous solution for the next adsorption. In practical safe disposal applications, after a certain concentration of radioactive TcO_4^-/ReO_4^- elements are reached in the eluate, the waste liquid is physically sealed, e.g. vitrified.

Scale-up tests of iPAF-P67/PES beads and nanofibers. For the flow test of beads-filled columns, two 200 g commercial adsorbent columns were connected in series with a 1.5 g iPAF-P67 beads column. The initial feed solution concentration was set to 22.5 ppm. The feed flow rate was controlled by peristaltic pump and stabilized at 0.5 mL/min. For the penetration test of nanofibers, the as-developed iPAF-P67/PES nanofibers were sandwiched on an H-shaped test cell and the adsorption test process was run under gravity drive. The initial feed solution concentration was set to 50 ppb. In dynamic adsorption tests, to ensure the proper functioning of the peristaltic pump, the simulated solution was filtered through a 0.22 μm aqueous filter membrane to remove solid impurities.

5. Computational method.

To better understand the excellent adsorption properties of iPAF-P67 toward TcO_4^-/ReO_4^-, the positively charged local structures of the as-developed iPAF-67, iPAF-P67 and the commercial

resin Purolite A530E were modeled by the A $[C_3N_2H_3\text{-}CH_2\text{-}C_6H_5\text{-}C_3N_2H_3\text{-}CH_2]^{2+}$ fragment, A' $[C_3N_2H_3\text{-}C_2H_4\text{-}C_3N_2H_3]^{2+}$ fragment and the B $[C_6H_5\text{-}CH_2\text{-}R_4N]^{+}$ fragment, respectively. Geometry optimizations were performed with the M062X functional with D3 version of Grimme's dispersion corrections[6, 7]. The def2-SVP basis set was employed for all atoms[8]. All of the calculations were performed with Gaussian 09[9]. The quantitative analysis of electrostatic potential on van der Waals (vdW) surface was performed using the Multiwfn 3.8 program[10]. Additionally, single point energy calculations were performed with the M062X-D3 functional, and the def2-TZVP for all atoms[11], with the inclusion of solvation energy corrections based on SMD implicit solvent model with water as solvent ($\varepsilon = 78.3553$)[12]. All the reported enthalpy values are the sum of the electronic energy from the single point calculations and the thermal correction to enthalpy obtained by the frequency calculations.

Section S2. Supplementary Figures and Tables

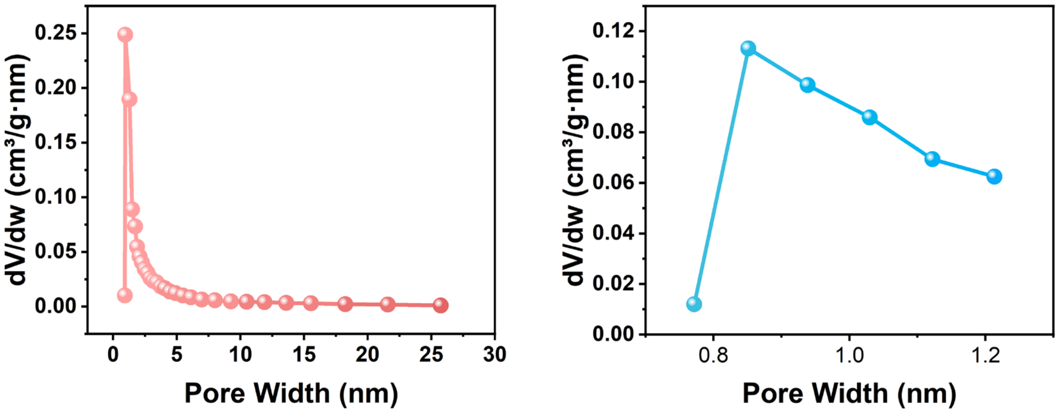

(a) (b)

Figure S1. Pore size distribution of iPAF-67 (a) and iPAF-P67 (b).

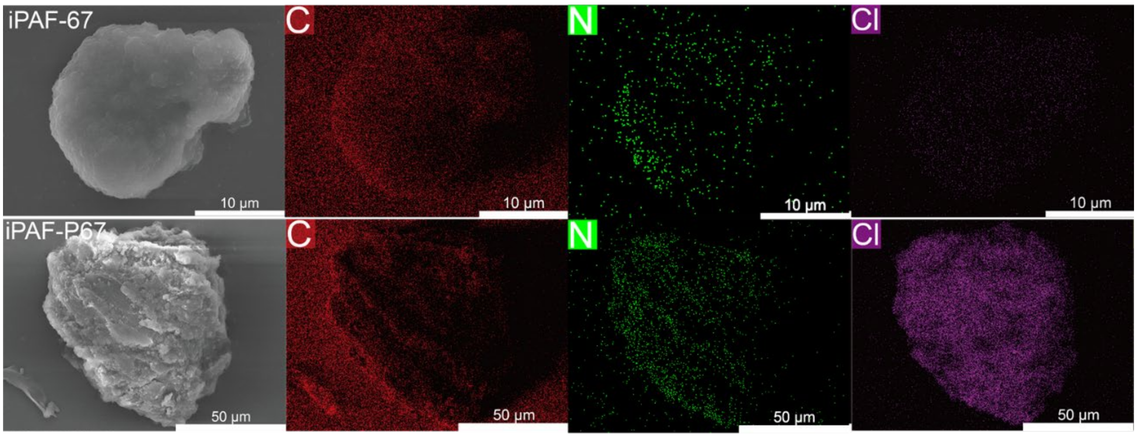

Figure S2. SEM and EDS-mapping images of iPAF-67 (upper) and iPAF-P67 (bottom).

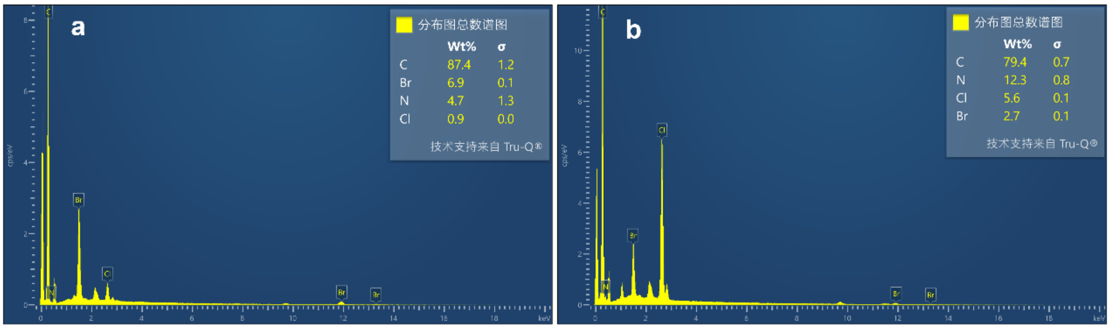

Figure S3. Element composition of iPAF-67 (a) and iPAF-P67 (b) based on SEM-EDS images. The content of imidazolium groups is calculated by dividing the mass percentage content of nitrogen (wt%) by the relative atomic mass of nitrogen (14.007). The content of the imidazolium groups is calculated based on the halogen or nitrogen content. Since some bromide ions in the material cannot be completely exchanged by chloride ions, and only the imidazolium groups contain nitrogen, the content of the imidazolium groups is consistent with the nitrogen content. The calculation formula is as follows:

$$C_{imidazolium} = \frac{wt_N\%}{M_N} \times 10^3 \text{ mmol/g} \qquad (7)$$

where $wt_N\%$ and M_N represent mass percentage of elemental nitrogen and molar mass of elemental nitrogen, respectively.

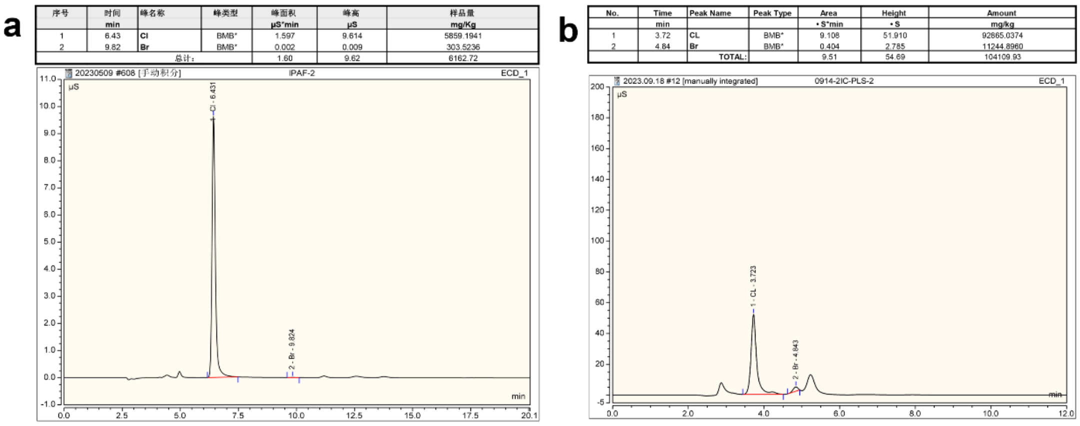

a

序号	时间	峰名称	峰类型	峰面积	峰高	样品量
	min			µS*min	µS	mg/Kg
1	6.43	**Cl**	BMB*	1.597	9.614	5859.1941
2	9.82	**Br**	BMB*	0.002	0.009	303.5236
总计：				1.60	9.62	6162.72

b

No.	Time	Peak Name	Peak Type	Area	Height	Amount
	min			• S*min	• S	mg/kg
1	3.72	**CL**	BMB*	9.108	51.910	92865.0374
2	4.84	**Br**	BMB*	0.404	2.785	11244.8960
		**TOTAL:**		9.51	54.69	104109.93

Figure S4. Ion chromatography for identifying halogen in iPAF-67 (a) and iPAF-P67 (b).

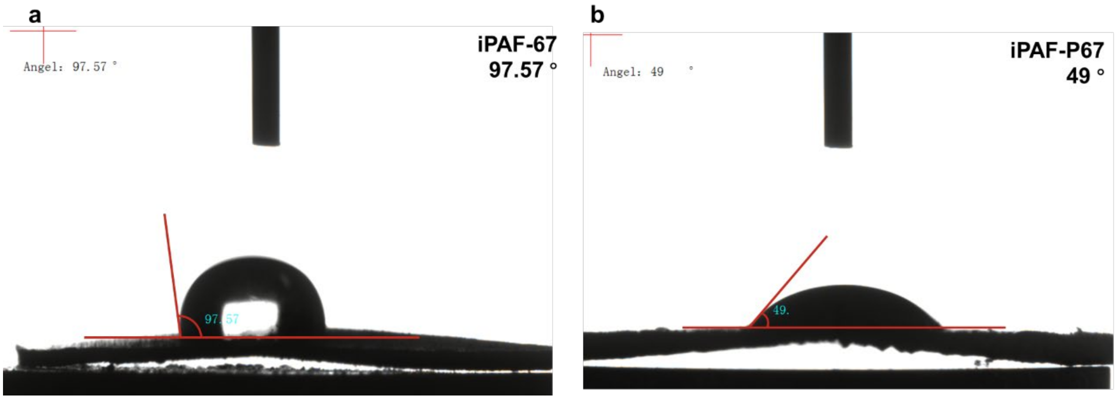

Figure S5. Water contact angles of iPAF-67 (a) and iPAF-P67 (b).

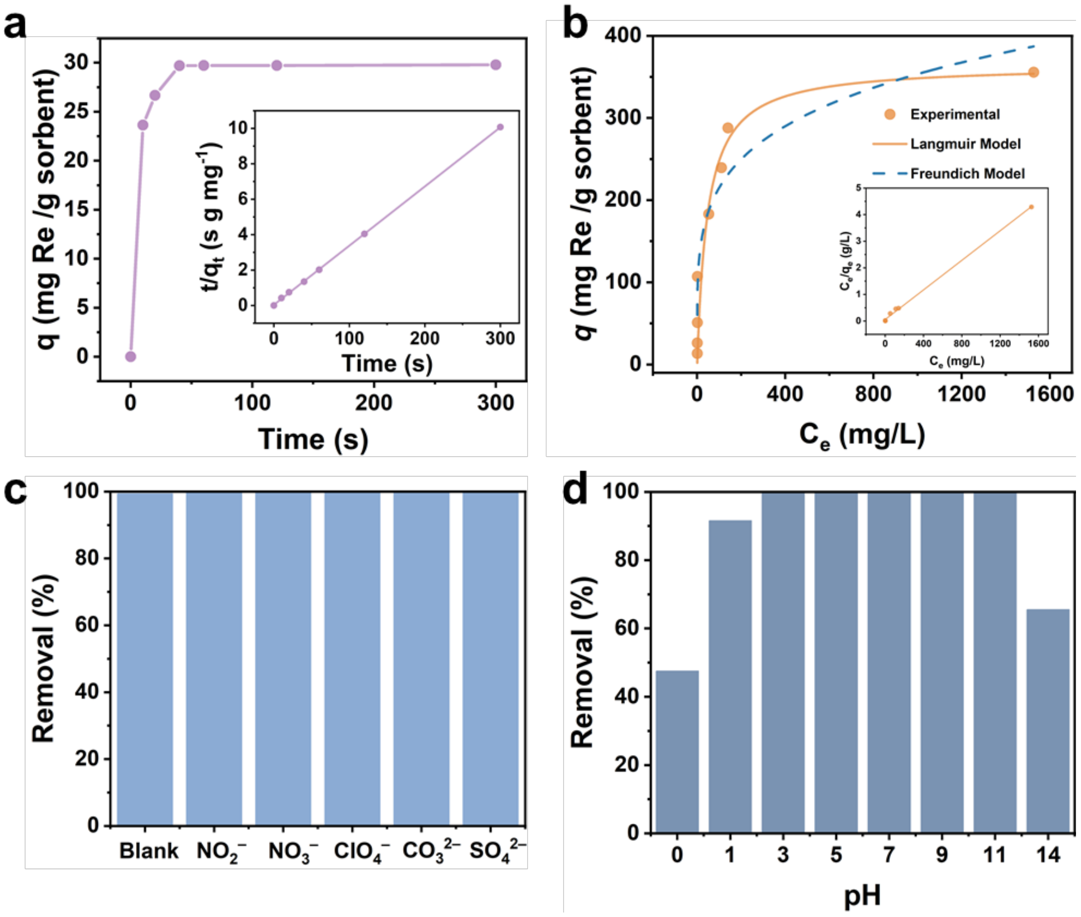

Figure S6. Adsorption performance of iPAF-67. (a) Adsorption kinetics of iPAF-67 toward ReO_4^- and its pseudo-second-order linear fitting plots (Inset). (b) Adsorption isotherm of iPAF-67 for ReO_4^- uptake. (c) Removal efficiency of ReO_4^- by iPAF-67 in the presence of competitive anions. (d) Effect of pH on the ReO_4^- sorption performances of iPAF-67.

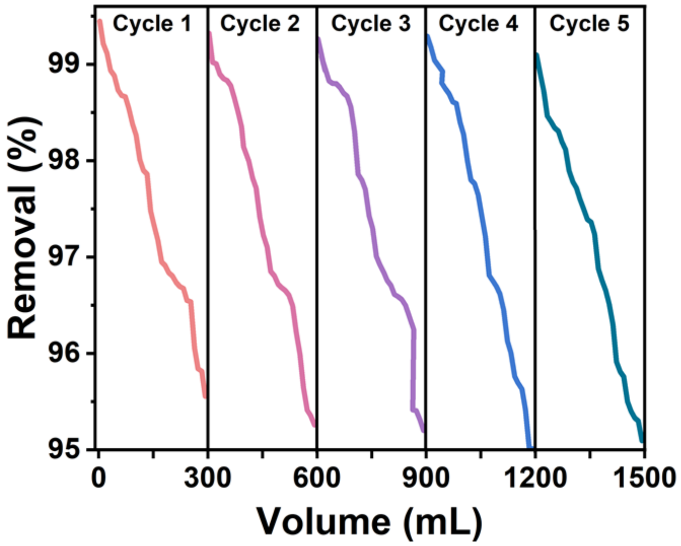

Figure S7. ReO_4^- removal efficiency of iPAF-P67 in five consecutive adsorption–desorption cycles.

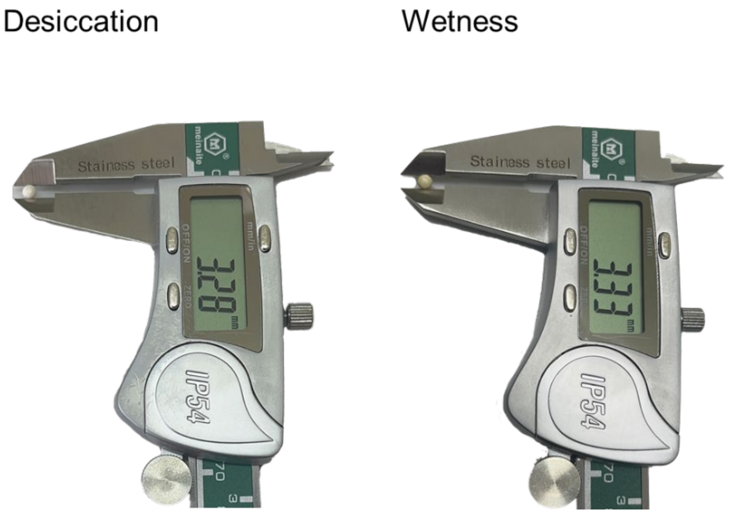

Figure S8. Diameter change of iPAF-P67 before and after adsorption test.

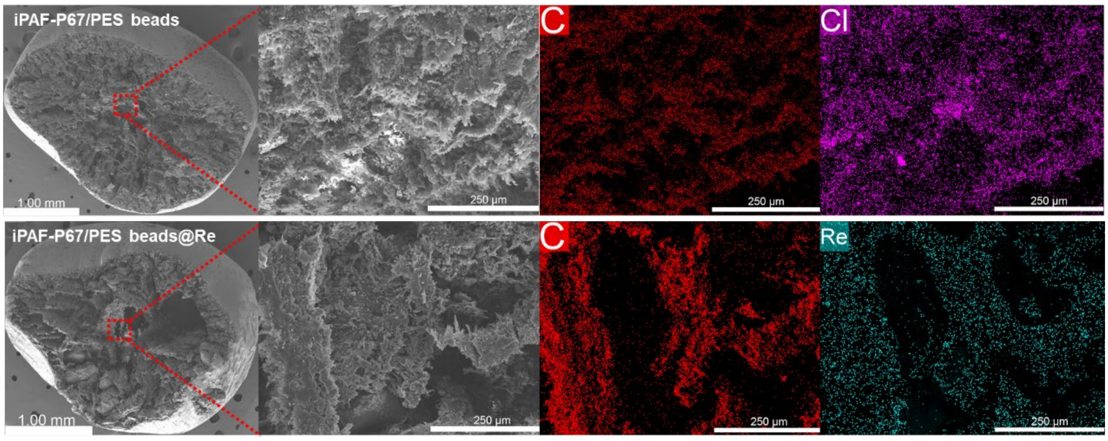

Figure S9. Morphologies and element changes of iPAF-P67/PES beads before and after adsorption test.

Table S1. Ion chromatography data for identifying halogen in iPAF-67 and iPAF-P67.

Name	Peak Name	Peak Type	Area S*min	Height S	Amount mg/L
iPAF-67	Cl	BMB *	1.597	9.614	5859.194
iPAF-P67	Cl	BMB *	9.108	51.910	92865.037

Table S2. Model parameters and correlation coefficient for ReO_4^- sorption kinetics by iPAF-67 and iPAF-P67.

**Samples**	**kinetics**			
	q_e (mg g^{-1})	k_2 (g mg^{-1} min^{-1})	R^2	Removal (%)
iPAF-67	29.92	1.75	> 0.9999	>99.0%
iPAF-P67	29.81	10.13	> 0.9999	>99.0%

Table S3. Fitting results of ReO_4^- sorption by iPAF-67 and iPAF-P67 based on the Langmuir and Freundlich models.

**Samples**	**Langmuir**			**Freundlich**		
	q_m (mg Re/g sorbent)*	k_L (L mg^{-1})	R^2	K_F (L^n/mol^{n-1}g)	n	R^2
iPAF-67	355.78	0.021	0.88	79.71	4.64	0.90
iPAF-P67	978.73	0.064	0.99	185.07	4.04	0.91

* The maximum adsorption capacities of iPAF-67 and iPAF-P67 for ReO_4^- are calculated to be 478 and 1317 mg·g^{-1}, respectively.

Table S4. Anion concentration and molar ratio of Hanford low activity waste (LAW).

Anion	Concentration (mol L^{-1})	Anion: ReO_4^- (molar ratio)
TcO_4^-/ReO_4^-	1.94×10^{-4}	1.0
NO_3^-	6.07×10^{-2}	314
Cl^-	6.39×10^{-2}	330
NO_2^-	1.69×10^{-1}	873
SO_4^{2-}	6.64×10^{-6}	0.343
CO_3^{2-}	4.30×10^{-5}	0.222

Table S5. Anion concentration and molar ratio of simulated legacy nuclear wastes in Savannah River Site (SRS).

Anion	Concentration (mol L^{-1})	Anion: ReO_4^- (molar ratio)
TcO_4^-/ReO_4^-	7.92×10^{-5}	1.0
NO_3^-	2.6	32819
OH^-	1.33	16788
NO_2^-	1.34×10^{-1}	1691
SO_4^{2-}	5.21×10^{-1}	6576
CO_3^{2-}	2.6×10^{-2}	328

Section S3. References

[1] C.-P. Li, H.-R. Li, J.-Y. Ai, J. Chen, M. Du, *ACS Cent. Sci.* **2020**, *6*, 2354–2361.

[2] N. Shen, Z. Yang, S. Liu, X. Dai, C. Xiao, K. Taylor-Pashow, D. Li, C. Yang, J. Li, Y. Zhang, M. Zhang, R. Zhou, Z. Chai, S. Wang, *Nat. Commun.* **2020**, *11*, 5571.

[3] J. Li, X. Dai, L. Zhu, C. Xu, D. Zhang, M. A. Silver, P. Li, L. Chen, Y. Li, D. Zuo, H. Zhang, C. Xiao, J. Chen, J. Diwu, O. K. Farha, T. E. Albrecht-Schmitt, Z. Chai, S. Wang, *Nat. Commun.* **2018**, *9*, 3007.

[4] a) Q. Sun, L. Zhu, B. Aguila, P. K. Thallapally, C. Xu, J. Chen, S. Wang, D. Rogers, S. Q. Ma, *Nat. Commun.* **2019**, *10*, 1646; b) Z. Di, Z.-F. Liu, H.-R. Li, Z. Liu, C.-P. Li, *Inorg. Chem. Front.* **2023**, *10*, 952–958.

[5] a) Y. Zhao, D. G. Truhlar, *Theor. Chem. Acc.* **2008**, *120*, 215-241; b) S. Grimme, J. Antony, S. Ehrlich, H. Krieg, *J. Chem. Phys.* **2010**, *132*, 154104.

[6] Y. Matsuo, E. Nakamura, *Organometallics* **2003**, *22*, 2554-2563.

[7] M. Frisch, G. Trucks, H. B. Schlegel, G. E. Scuseria, M. Robb, J. R. Cheeseman, G. Scalmani, V. Barone, G. A. Petersson, H Nakatsuji, X. Li, M Caricato, A. Marenich, J Bloino, Benjamin G. Janesko, R Gomperts, B. Mennucci, H. Hratchian, J. V. Ortiz, A. izmaylov, J. Sonnenberg, D. Williams-Young, F. Ding, F. Lipparini, F. Egidi, J Goings, B. Peng, A. Petrone, T. HENDERSON, D Ranasinghe, Vyacheslav G. Zakrzewski, J. Gao, N Rega, G ZHENG, W. Liang, M Hada, M. EHARA, K. TOYOTA, R. FUKUDA, J Hasegawa, M. ISHIDA, T. NAKAJIMA, Y. Honda, O. Kitao, H NAKAI, T. Vreven, K. Throssell, J. Montgomery, J. Peralta, F. Ogliaro, M. Bearpark, J. J Heyd, E. Brothers, K. Kudin, V. Staroverov, T. Keith, R Kobayashi, J. Normand, K. RAGHAVACHARI, A. P. Rendell, J. C. Burant, S. Iyengar, J. TOMASI, M Cossi, J. M. Millam, M Klene, C Adamo, R. Cammi, J. W. Ochterski, R. MARTíN, K Morokuma, O. Farkas, J. Foresman, D. Fox, G. Trucks, G. Scalmani, G. A. Petersson, J. Bloino, R. Gomperts, B. MENNUCCI, H. Hratchian, J. V. Ortiz, A. Izmaylov, J. Sonnenberg, J. Goings, T Henderson, Viatcheslav G. Zakrzewski, J. GAO, N. Rega, G. Zheng, M. Ehara, J. HASEGAWA, M. Ishida, T. Nakajima, Y. HONDA, T Vreven, K. Throssell, J. Peralta, E. Brothers, R. KOBAYASHI, K. Raghavachari, J. Tomasi, R. CAMMI, R. A. Martin, J. B. FORESMAN, D. Fox, H. B. Schlegel, A. Izmaylov, F Lipparini, B Peng, G Zheng, W. LIANG, R Fukuda, M Ishida, O Kitao, H. Nakai, J. A. Montgomery Jr, F Ogliaro, M. Bearpark, V. Staroverov, R. Kobayashi, M. Cossi, C Adamo, R. D. MARTIN, J. Foresman, M Frisch, G Trucks, H. Schlegel, G Scuseria, M. Robb, J Cheeseman, G PETERSSON, X Li, A. Marenich, B Janesko, H Hratchian, J Ortiz, A. Izmaylov, J Sonnenberg, F Ding, T. Henderson, V.

Zakrzewski, M Hada, J. MONTGOMERY, J Peralta, M. Bearpark, J Heyd, E BROTHERS, K. Kudin, V Staroverov, T Keith, A Rendell, J. Burant, S Iyengar, J Tomasi, J Millam, M. Klene, J Ochterski, R. MARTIN, O Farkas, J. Foresman, D. FOX, M. Frisch, V. BARONE, A Petrone, W Liang, Y. Honda, H Nakai, J. C Burant, S. IYENGAR, J. A. Montgomery Jr., J. Millam, J. Knox, J. N CROSS, V Bakken, J Jaramillo, R. E. Stratmann, O Yazyev, A. Austin, C Pomelli, G. Voth, P Salvador, J. J. Dannenberg, S Dapprich, A. Daniels, J Cioslowski, J Gao, J. J. Heyd, K Toyota, T Nakajima, J. Montgomery, K Raghavachari, J. Frisch M., J. Peralta, K. A. Kudin, ALISTAIR RENDELL, J. Cross, V. BAKKEN, J. JARAMILLO, V. Barone, Alistair Rendell, J. V. O. iz, WILLIAMS, J. A. Montgomery, J. Zheng, E Brothers, K. N. Kudin, J. C. T. Rendell, S. Burant, A. AUSTIN, P SALVADOR, M.J. Frisch, W. Li, J. Montgomery, Jr., MJ Frisch, GW Trucks, HB Schlegel, GE Scuseria, MA Robb, JR Cheeseman, GA Petersson, AV Marenich, BG Janesko, HP Hratchian, JV Ortiz, AF Izmaylov, JL Sonnenberg, VG Zakrzewski, J J. A. Montgomery, JE Peralta, MJ Bearpark, JJ Heyd, EN Brothers, KN Kudin, VN Staroverov, TA Keith, AP Rendell, JC Burant, SS Iyengar, JM Millam, JW Ochterski, RL Martin, JB Foresman, DJ Fox, Gaussian 16, Revision A.03, *Gaussian, Inc,* Wallingford CT, **2016**.

[8] T. Lu, F. Chen, *J. Comput. Chem.* **2012**, *33*, 580–592.

[9] F. Weigend, R. Ahlrichs, *Phys. Chem. Chem. Phys.* **2005**, *7*, 3297–3305.

[10] A. V. Marenich, C. J. Cramer, D. G. Truhlar, *J. Phys. Chem. B* **2009**, *113*, 6378–6396.
